# A low‐cost protocol for the optical method of vulnerability curves to calculate *P*_50_

**DOI:** 10.1002/aps3.70004

**Published:** 2025-03-31

**Authors:** Georgina González‐Rebeles, Miguel Ángel Alonso‐Arevalo, Eulogio López, Rodrigo Méndez‐Alonzo

**Affiliations:** ^1^ Departamento de Biología de la Conservación, Centro de Investigación Científica y de Educación Superior de Ensenada Carretera Ensenada Tijuana No. 3918, Zona Playitas, C.P. 22860 Ensenada Baja California Mexico; ^2^ Departamento del Hombre y su Ambiente Universidad Autónoma Metropolitana–Unidad Xochimilco Calzada del Hueso 1100, Colonia Villa Quietud, Alcaldía Coyoacán, C.P. 04960 Ciudad de México Mexico; ^3^ Departamento de Electrónica y Telecomunicaciones, Centro de Investigación Científica y de Educación Superior de Ensenada Carretera Ensenada Tijuana No. 3918, Zona Playitas, C.P. 22860 Ensenada Baja California Mexico

**Keywords:** cost‐effective protocols, image processing, percentage of embolisms accumulated, stem water potential, xylem tissue, curvas de vulnerabilidad, método óptico, porcentaje de embolismos acumulados, potencial hídrico del tallo, tejido del xilema, procesamiento de imágenes

## Abstract

**Premise:**

The quantification of plant drought resistance, particularly embolism formation, within and across species, is critical for ecosystem management and agriculture. We developed a cost‐effective protocol to measure the water potential at which 50% of hydraulic conductivity (*P*_50_) is lost in stems, using affordable and accessible materials in comparison to the traditional optical method.

**Methods and Results:**

Our protocol uses inexpensive USB microscopes, which are secured along with the plants to a pegboard base to avoid movement. A Python program automatized the image acquisition. This method was applied to quantify *P*_50_ in an exotic species (*Nicotiana glauca*) and native species (*Rhus integrifolia*) of the Mediterranean vegetation in Baja California, Mexico.

**Conclusions:**

The intra‐ and interspecific patterns of variation in stem *P*_50_ of *N. glauca* and *R. integrifolia* were obtained using the low‐cost optical method with widely available and affordable materials that can be easily replicated for other species.

## Resumen

**Premisa:**

La cuantificación inter e intraespecífica de la resistencia de las plantas a la sequía, mediante la formación de embolismos, es fundamental para el manejo de ecosistemas y agricultura. Desarrollamos un método rentable para medir el potencial hídrico en el que se pierde 50% de la conductividad hidráulica (*P*_50_) en tallos, utilizando materiales asequibles y accesibles en comparación con el método óptico tradicional.

**Métodos y resultados:**

Nuestro protocolo consiste en utilizar microscopios USB económicos que se aseguran con plantas en una base perforada para evitar su movimiento. Automatizamos con Python la adquisición de imágenes y cuantificamos *P*_50_ en una especie exótica (*Nicotiana glauca*) y una nativa (*Rhus integrifolia*) de la vegetación mediterránea en Baja California, México.

**Conclusiones:**

Los patrones intra e interespecíficos de variación en el *P*_50_ de *N. glauca* y *R. integrifolia* se obtuvieron con el método óptico con materiales disponibles y asequibles y puede replicarse fácilmente con otras especies.

Water stress strongly affects the productivity of natural and agricultural ecosystems. It can be considered the precursor to plant mortality, as it triggers a systemic cascade of physiological responses that severely constrain carbon acquisition and metabolite production (McDowell et al., 2008). Some species are more drought resistant than others (Choat et al., 2012), however, and understanding the drought resistance of various native, exotic, and cultivated species allows us to better predict species distribution in natural areas and select more resistant species or varieties for restoration or agricultural projects (Engelbrecht et al., 2007). Despite the importance of quantifying water stress, it has been challenging to identify functional attributes that are both directly related to plant mortality due to drought and can be quickly and easily measured.

Plant drought and hydraulic attributes are explained by the tension–cohesion mechanism of water transport (Angeles et al., 2004; McDowell et al., 2019). In vascular plants, water is transported from the roots to the leaves primarily due to the tension generated by water loss through stomata and the cohesion provided by hydrogen bonds between water molecules and vessel walls (Jacobsen et al., 2005; Torres‐Ruiz et al., 2024a). As water is transported under highly negative pressures, a lack of water in the soil increases the tension, leading to implosions and malfunctions in the xylem. These conditions can cause changes in gas dissolution (i.e., cavitation) and, consequently, the formation of air bubbles (i.e., embolisms) that block water passage (Jacobsen et al., 2005; Torres‐Ruiz et al., 2024a). Over time, this blockage can induce plant mortality. The vulnerability to embolism formation is directly associated with various plant ecological and physiological properties, even affecting the biogeographical distribution of biodiversity (Blackman et al., 2012; Choat et al., 2018).

Due to its significance, many physiologists have sought efficient ways to measure the water potential that causes 50% of hydraulic conductivity (*P*_50_) to be lost (Sperry et al., 1988; Brodribb et al., 2017; Nardini et al., 2017). *P*_50_ is considered a valuable comparative variable in physiological studies and is mainly derived from vulnerability curves that plot plant water potential during desiccation on the *x*‐axis against the percentage of hydraulic conductivity loss on the *y*‐axis (Choat et al., 2012). The methods for generating vulnerability curves vary in cost and time and can be classified according to the desiccation process and the conductivity loss measurement process (Venturas et al., 2019). There are three main desiccation processes: natural desiccation (bench drying), desiccation through a pressurized gas application (air injection), and embolism formation using a centrifuge. The centrifuge method can be expensive and inaccessible for certain projects, and pressurized gas can damage vascular tissue (Chen et al., 2021). Therefore, the most accessible and recommended method for dehydration is the bench dry method (Venturas et al., 2019).

Conductivity loss measurement can be either hydraulic or visual. Hydraulic processes measured through a conductivity apparatus are the most direct way to assess conductivity loss (Sperry et al., 1988). However, this method requires cutting plants into specific sections, which can introduce air and cause disturbances in the plant (Brodribb et al., 2017), and the labor involved can be time consuming. Visual methods include techniques such as microtomography (micro‐CT), which allows 3D visualization of xylem vessels, distinguishing between conductive and non‐conductive tissue; however, this requires very expensive equipment and software (Venturas et al., 2019; Cardoso et al., 2022). The optical method, initially developed by Brodribb et al. (2016a, 2017), is a relatively new visual technique that allows for the visualization of embolism formation. This method tracks embolism formation in different plant organs by detecting changes in light transmission through vessels, due to the contrast between water‐filled and air‐filled vessels (Cardoso et al., 2022). This change in light transmittance can be observed through microscope‐enhanced time‐lapse photography of exposed xylem tissues (Brodribb et al., 2017). Stereo microscopes or cameras—secured to plant organs with additional material—are typically used, but their high cost makes these inaccessible to many researchers (Table 1). Petruzzellis et al. (2020) developed a cheap and accessible method to apply the optical method to leaf laminas using smartphone cameras; however, despite such advances, there has been little discussion on developing more affordable optical methods to measure *P*_50_ in stems.

**Table 1:** Different hardware used for the optical method to determine the water potential that causes 50% of hydraulic conductivity loss in stems (*P*_50_), the brand name of the equipment, the estimated cost, and the references for the studies in which the hardware was used.

Hardware	Brand name	Estimated cost^a^	References
Scanner	Epson Perfection V800 or V850	$315.00	Skelton et al. (2017, 2019); Lucani et al. (2019); Creek et al. (2020); Levionnois et al. (2020); Song et al. (2022)
Scanner	CanoScan 8800F transparent film scanner (Canon)	$134.17	Brodribb et al. (2016a)
Stereoscopic microscope with digital camera	Leica M205 stereoscopic microscope; Leica DFC450 digital camera; Optika S2MTZ stereo microscope	$1828.91	Brodribb et al. (2016b, 2017); Venturas et al. (2019); Chen et al. (2021); Guan et al. (2021)
Stereoscopic microscope	AxioZoom V16 stereo microscope (Zeiss)	$1932.43	Guan et al. (2021)
Stereoscopic microscope	Optika S2MTZ stereo microscope	$596.72	Avila et al. (2021, 2023)
Camera and lens	Raspberry Pi 8 MP camera; magnification lens 30×; stem clamp to avoid movement (Raspberry Pi)	$798.11	Rodriguez‐Dominguez et al. (2018); Smith‐Martin et al. (2020); Guan et al. (2021); Tomasella et al. (2021); Avila et al. (2021, 2023); Johnson et al. (2022); Lucani (2024)
Camera and macrolens	Fujifilm XT‐2 digital camera; Mitakon Creator 20 mm f/2 macrolens	$999.00 + 890.16	Venturas et al. (2019); Pratt et al. (2020)
Smartphone	Android OS (ASUS smartphone)	$299.00	Petruzzellis et al. (2020)

^a^ Costs are given in U.S. dollars (USD) based on the cost of new equipment at the time of acquisition.

We propose a new, accessible, and inexpensive approach using USB microscopes and a set of tools available at practically any hardware store to measure *P*_50_ in stems. Our goal is to implement this optical method with commercial, off‐the‐shelf USB microscopes in a broader range of contexts, particularly in the Global South, where much of the world's biodiversity is found. We validated our method by comparing the *P*_50_ between two contrasting woody species of the California Floristic Province, *Nicotiana glauca* Graham (Solanaceae) and *Rhus integrifolia* (Nutt.) Benth. & Hook.f. ex W.H.Brewer & S.Watson (Anacardiaceae), and then analyzed the costs and effectiveness of this new system.

## METHODS AND RESULTS

### Preparation of equipment

In January 2024, we acquired four microscope stands (Koolertron, Hong Kong Karstone Technology Co., Hong Kong, China; $19.99 USD per stand [costs based on exchange rates from the Organisation for Economic Co‐operation and Development (OECD), https://www.oecd.org/en/data/indicators/exchange-rates.html]) and four USB microscopes (generic USB microscope; $19.66 USD per microscope) from Amazon (Amazon.com, Seattle, Washington, USA). This low‐cost USB microscope is available under various brand names and from multiple vendors, but they all share the same fundamental features, including common image formats such as JPEG, PNG, or BMP, an integrated adjustable 8‐LED light source, a USB 2.0 connection interface, and manual focus capabilities within a range of 3–40 mm. The microscopes offer a color still image capture resolution of 0.3 megapixels (640 × 480) and a theoretical maximum magnification of 40×. Despite being sold under different labels, the core specifications remain consistent, providing an affordable and functional option for basic microscopy needs. Additionally, this type of microscope does not require any drivers to function on most operating systems, such as Windows, macOS, or Linux, because it complies with the USB video device class (UVC). This standard allows the microscope to be recognized by the computer as a video streaming device, like a webcam, enabling plug‐and‐play functionality across a wide range of platforms without the need for additional software. These microscopes are more cost‐effective than the original cameras used in the optical methods described by other authors (Table 1).

**Figure 1:**
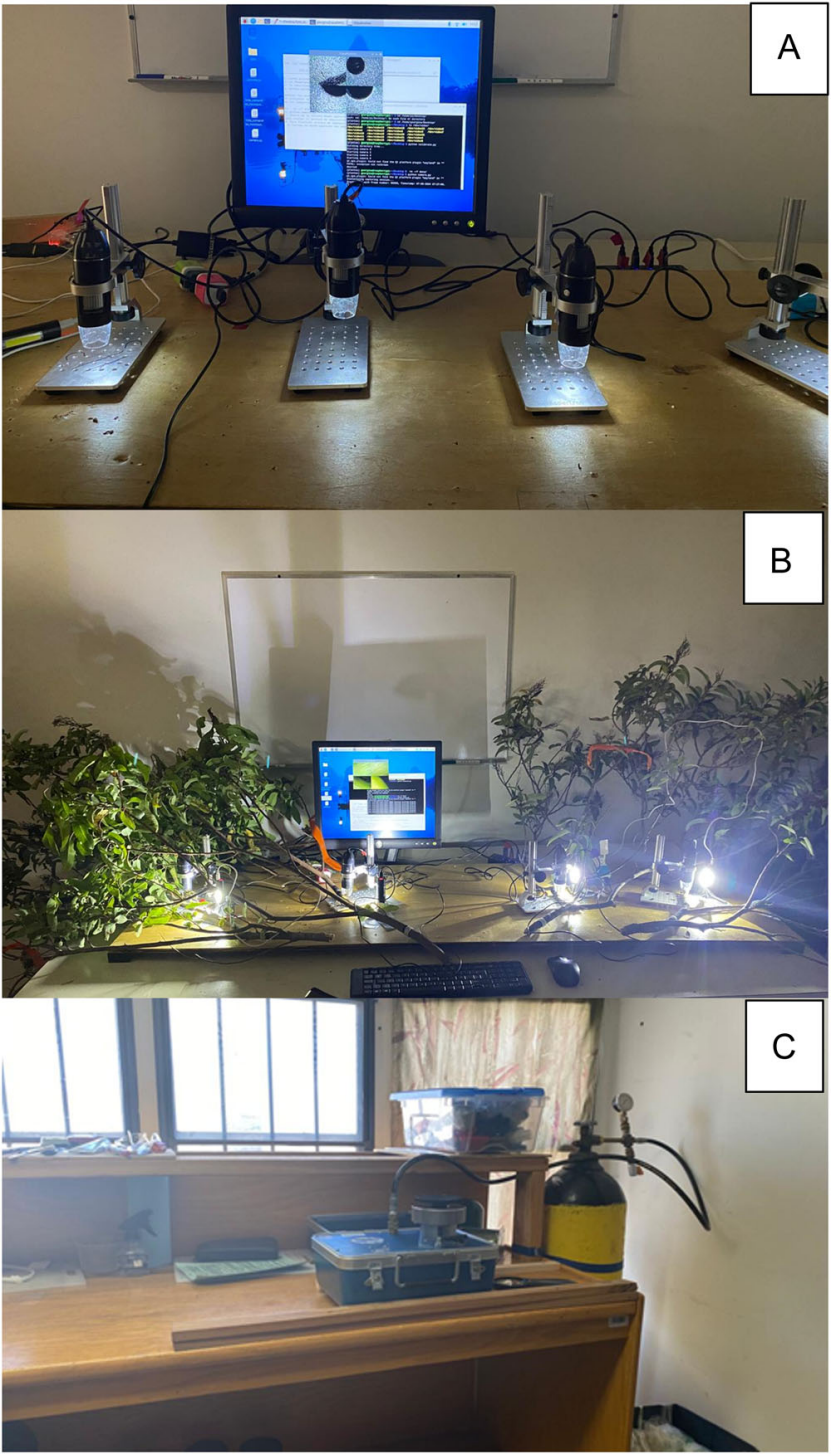
Illustration of the equipment setup for each stage of the protocol. (A) USB microscopes, perforated microscope pegboard stands, and the screen that was used for the experiment. (B) Preparation of the darkroom for the measurements of the percentage of embolisms. (C) Preparation of the outer room for the measurements of water potential. Photos taken at the Department of Conservation Biology, CICESE, Ensenada, Mexico, July–September 2024.

**Figure 2:**
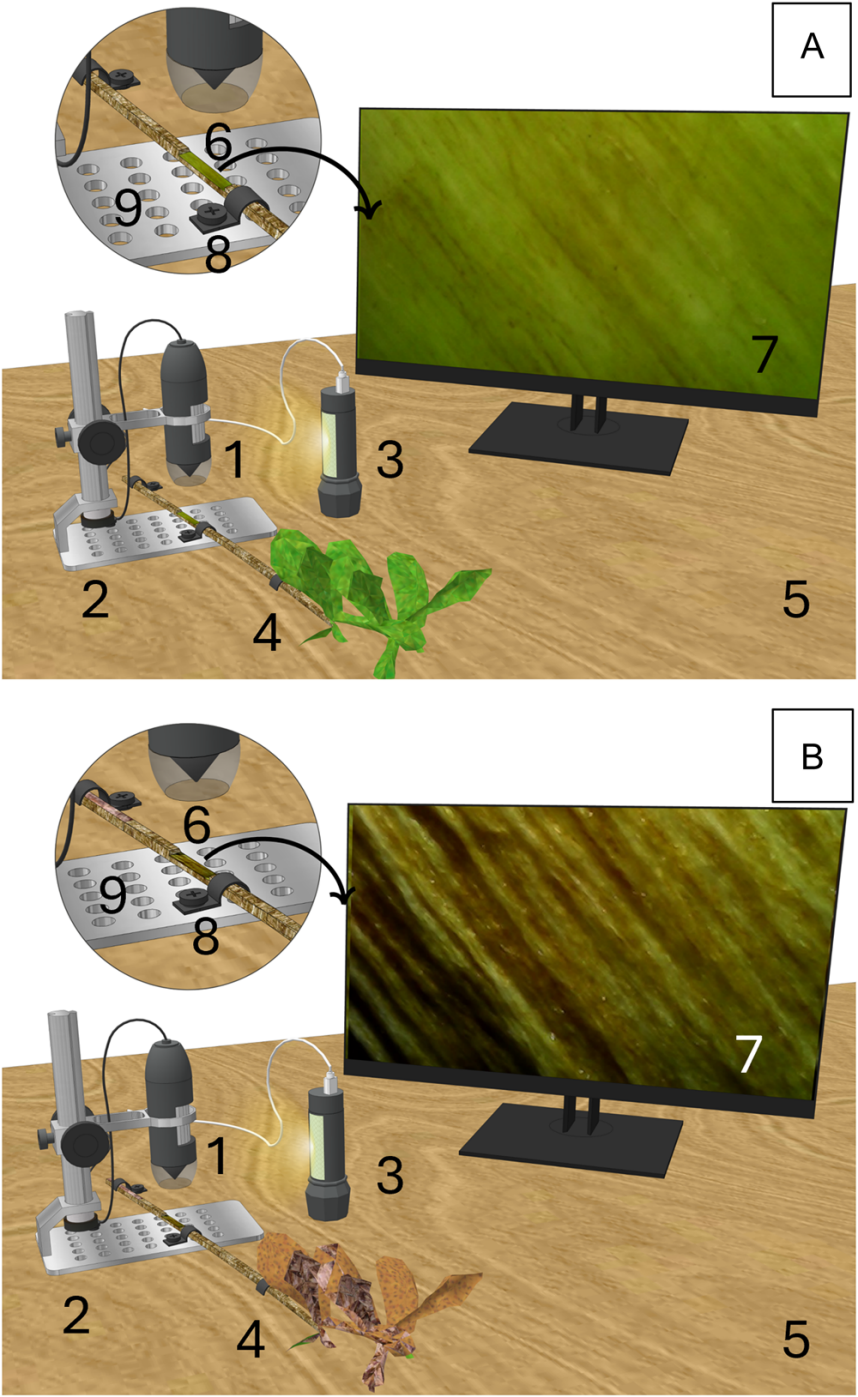
Diagram of the experiment with the fresh sample before cavitation induction (A) and the dry sample after cavitation induction (B), showing all the components used: (1) USB microscope, (2) microscope stand, (3) rechargeable LED lantern, (4) cut stem, (5) plywood, (6) small window of exposed xylem, (7) exposed xylem as seen by the USB microscope, (8) wire clips and screws, and (9) perforated microscope stand. Illustration by Rosa Lilia Pérez Arce.

We cut a 160‐cm‐long and 50‐cm‐wide piece of ½‐inch birch plywood ($32.83 USD). Additionally, we purchased nylon plastic R‐type wire clips and screws of various sizes ($9.99 USD for 268 pieces) to facilitate the fixation of branches and minimize movement during photo capture. Perforations were made in both the plywood and the base of the microscope stands (Figures 1A and 2) to secure the microscopes onto the wooden surface. The wire clips and screws were used to hold the stems in place on the wood, close to the microscope stands.

To minimize reflections from bright surfaces, such as plant tissue with high water or moisture content, adjustments in the lighting angle are necessary. Therefore, we turned off the integrated light in the microscope and instead used a more diffused external light source (EasyTao rechargeable LED lantern, EasyTao Comercializadora S.A. de C.V., Ciudad López Mateos, Mexico; $13.19 USD for four pieces). The light intensity on the stem section was 11.5 mW higher than in areas farther from the light. Despite the external light source being approximately 10.02°C above room temperature, the stem in contact with the light showed only a minimal temperature increase of 0.4°C compared to room temperature (Appendix S1). The setup of all the equipment is illustrated in Figures 1 and 2.

**Figure 3:**
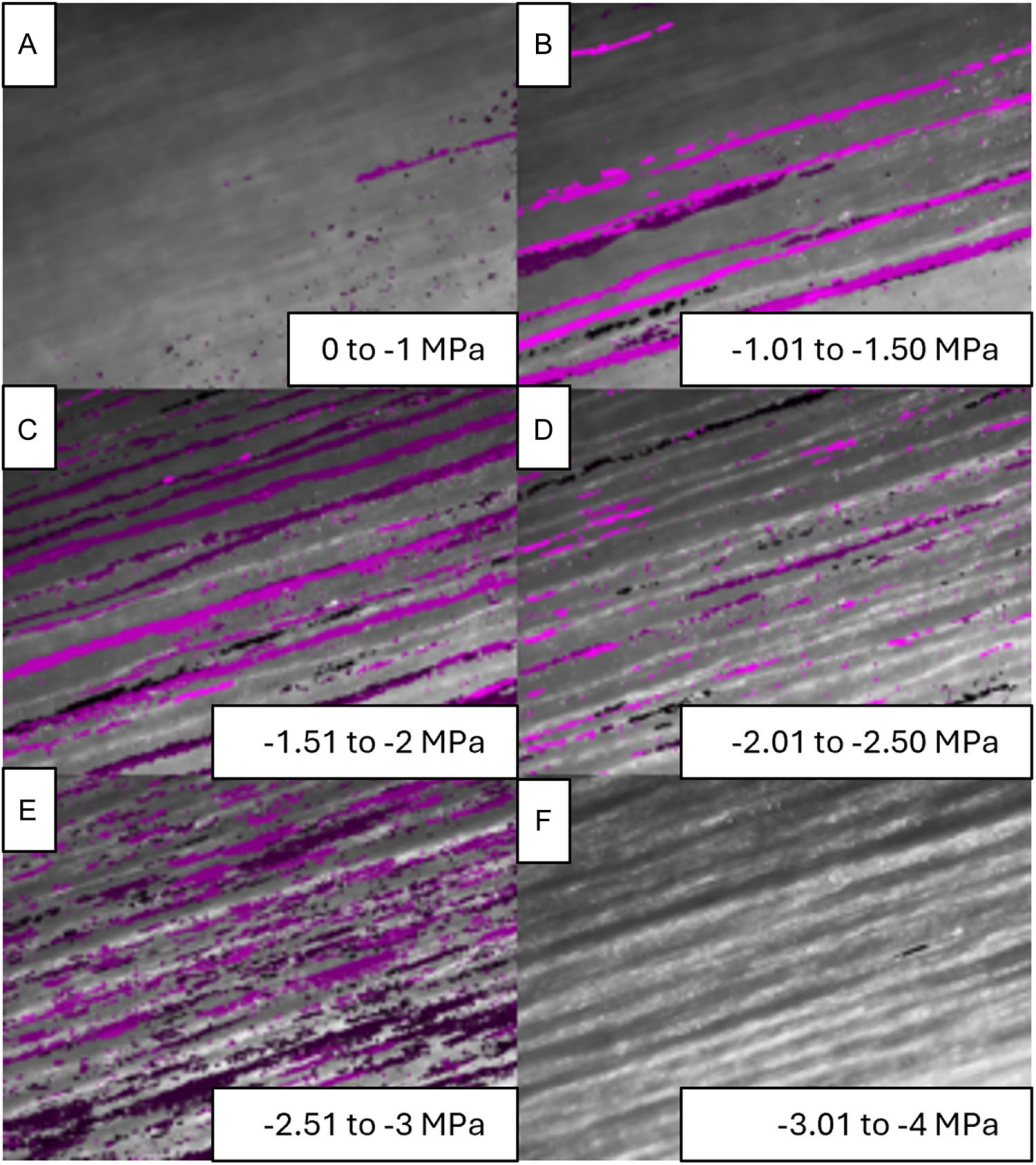
Images showing embolism formation in the xylem of *Nicotiana glauca*. The gradient of black to magenta lines indicates the embolisms formed during each interval of water potential. (A) 0 to −1 MPa, (B) −1.01 to −1.50 MPa, (C) −1.51 to −2 MPa, (D) −2.01 to −2.50 MPa, (E) −2.51 to −3 MPa, (F) −3.01 to −4 MPa.

**Figure A3:**
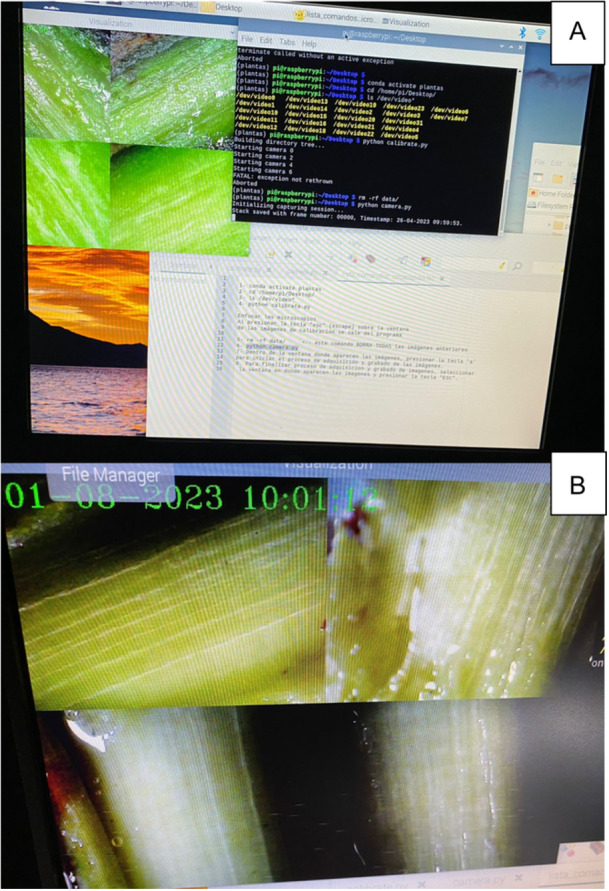
Images of the screen showing the activated program. (A) In the upper left corner, images from each of the four microscopes are displayed, and on the right, the text output indicates the status. (B) Images from each of the four microscopes are displayed in a larger view.

A Python program was developed to enable the simultaneous capture of photos from all four microscopes every four minutes (available at: https://github.com/miguel-aalonso/lowcost_P50; Appendices S2 and S3). The program also allows for the simultaneous display and calibration of the observations from all microscopes (see Figure A3 in Appendix 1), and shows the most recent photos taken. The program saves the photos from each microscope into separate folders for individual analysis, with the time and date of each capture recorded. This timestamping is essential for posteriorly calculating embolism accumulation (Figure 3).

A darkroom was prepared to house the microscopes, ensuring minimal external interference from fluctuations in light intensity (see Figure 1A, B). Outside the darkroom, a table was arranged for water potential measurements (Figure 1C).

### Measurement of *P*_50_

**Figure 4:**
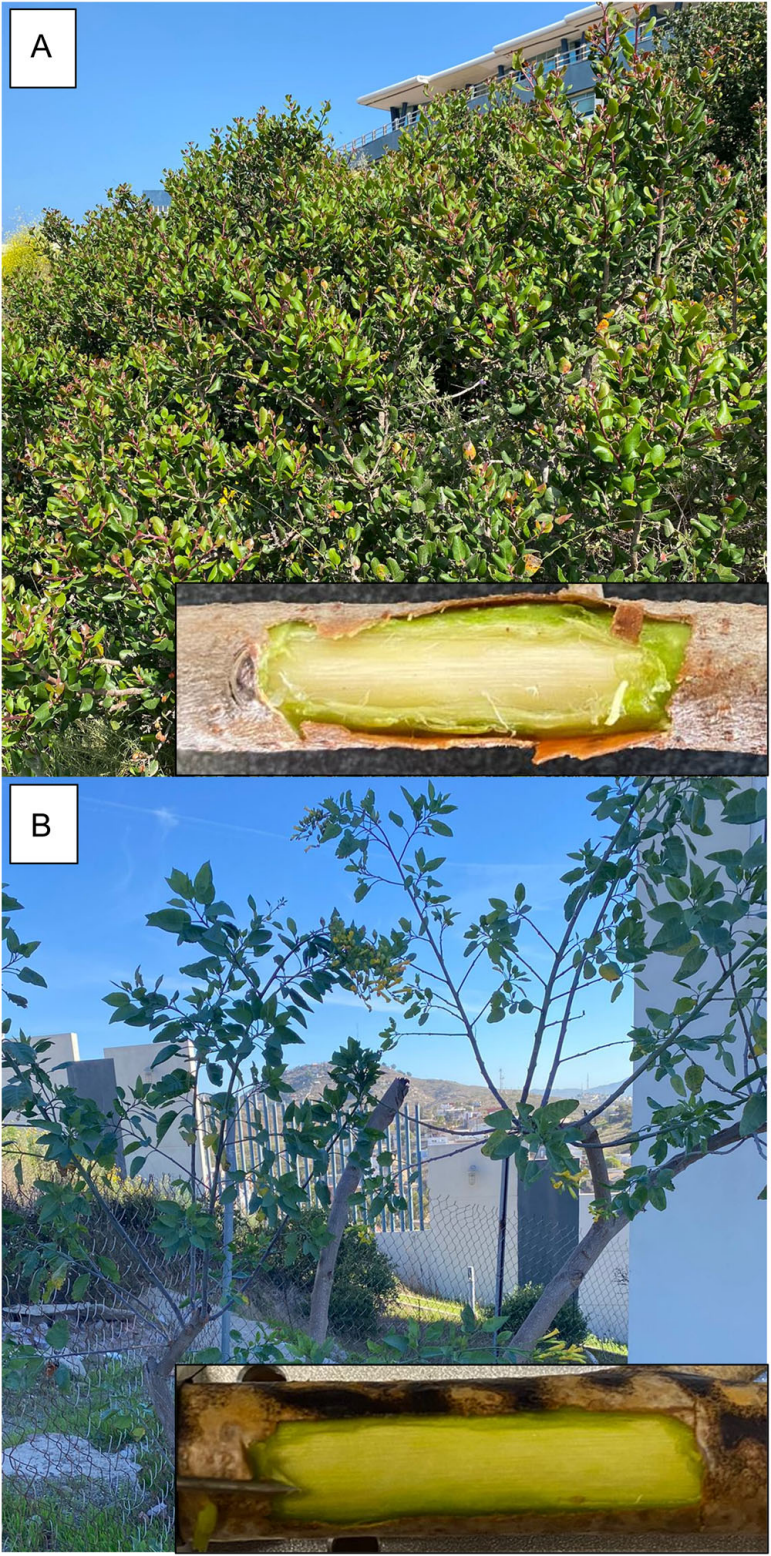
Individuals sampled for the study, with inset images of the exposed xylem of each species. (A) *Rhus integrifolia*, (B) *Nicotiana glauca*. Photos taken at the Department of Conservation Biology, CICESE, Ensenada, Mexico, July–September 2024.

Measurements of *P*_50_ were done on individuals of *Rhus integrifolia* (Figure 4A, data in Appendix S4), a locally dominant native species, and *Nicotiana glauca* (Figure 4B, data in Appendix S4), a widely distributed exotic species, at the Centro de Investigación Científica y de Educación Superior de Ensenada (CICESE) ecological reserve.

To estimate the adequate stem length to be collected for each species and to avoid the open‐vessel artifact (Sperry et al., 2012; Torres‐Ruiz et al., 2017), we measured the maximum xylem vessel length (*L*_*max*_) in six stems for each species using a modification of the Torres‐Ruiz et al. (2024b) protocol. The 12 stems were rehydrated for 24 hours in buckets of water covered with black bags. Each stem was connected to the outflow of a vacuum pump using plastic tubing of approximately the same diameter as the stems; the plastic tube was attached to the basal end of each stem, while the apical end was immersed in a bucket of water. The vacuum pump injected air at a pressure of up to 0.20 MPa and was applied continuously through the stem while progressively cutting 1‐cm portions from the apical to the basal end until a string of air bubbles appeared at the apical end. Garden shears were used to cut the apical region of the stem, and a magnifying glass with an LED lamp was used to observe the presence of air bubbles. The presence of air bubbles at the apical end indicated that a vessel had been cut on both sides of the stem. The length of the stem at the point where the first string of air bubbles appeared was recorded as the *L*_*max*_. For *R. integrifolia* and *N. glauca*, we obtained an *L*_*max*_ of 0.86 ± 0.046 m and 0.59 ± 0.043 m (average ± standard error), respectively (Appendix S5).

To estimate *P*_50_, terminal branches of branch length larger than *L*_*max*_ were gathered from seven individuals of *R. integrifolia* and *N. glauca* (Appendix S6). These branches were then rehydrated for 24 hours in buckets of water covered with black bags.

To expose the xylem tissue and remove the cortex and periderm, we used a cosmetology nail file (5″ Professional Sapphire File, metallic; $4.99 USD). The file was used to carefully expose the xylem tissue by filing away the hard part of the bark. Subsequently, the soft part of the phloem was manually removed, either with the blunt side of a small razor or with a fingernail, until the xylem was exposed (the xylem has a distinctive white‐yellowish color that sets it apart from the green phloem, as shown in Figure 4).

The exposed xylem was then covered with Tensive Conductive Adhesive Gel (Parker Laboratories, Fairfield, New Jersey, USA; $5.56 USD), following recommendations from M. Carins‐Murphy (Yale University) and T. Brodribb (University of Tasmania, personal communication) to prevent desiccation from light exposure and to enhance visibility under the microscope. To prevent the gel from running off the location, we only applied a small amount and reapplied it two days after the initiation of the experiment. The stems were then placed under the four microscopes in the darkroom, and image acquisition was initiated (see Figures 1B and 3). The temperature and relative humidity of the darkroom were 17°C and 52%, respectively (Appendix S1). The light intensity measured on the stems directly exposed to the light was 19.2 mW, while the intensity in the experimental setup away from the LED lamp was 10.6 mW. In contrast, the light intensity in the area of the room far from the experimental setup was 7.7 mW (Appendix S1).

The initial water potential measurement was taken at the same time as the first photo capture. To measure water potential, branches were bagged immediately after incision for 15 minutes, with moist paper towels placed inside each bag, following the recommendations of Rodriguez‐Dominguez et al. (2022). One branch was collected per sample, and an average was obtained from the four samples located in the four different microscopes. We used a PMS 1505D pressure chamber (PMS Instruments, Albany, Oregon, USA) for these measurements. Pressure chambers can be expensive, with a cost of approximately $4280 USD (Hydrocultura, 2024); this represents a significant limitation in financing the method, and it should be carefully considered when planning its application.

For the first three days, water potential was measured every six hours; thereafter, the maximum water potential was measured daily at 1200 hours (Figure 1C). After measuring water potential, we followed the methodology outlined by Lucani and Brodribb (2023) to estimate the water potential for each of the sequential photographs. Using Excel (Microsoft 365, Microsoft Corporation, Redmond, Washington, USA), we plotted the date and time against the water potential and calculated a linear regression equation. Next, we extracted the time stamps for each photograph and used the regression equation to estimate the corresponding water potential values. This approach allowed us to interpolate the water potential across all photographs, providing continuous data for constructing the vulnerability curves.

### Image analysis and statistics

To analyze the images and calculate the percentage of embolisms, we followed the methodology outlined by Lucani and Brodribb (2023) using Fiji software (Schneider et al., 2012; https://imagej.net/software/fiji/) (Figure 3). First, we processed the images using the OSOV toolbox developed by Lucani and Brodribb (2023), which estimates pixel changes between each photograph and its preceding one. We then manually excluded pixels that were not caused by embolisms and were likely due to movement. Next, we calculated the area of embolism‐related pixels for each photograph.

Using Excel, we plotted the timestamp of each photograph against the embolism area obtained. From these data, we calculated the accumulation of embolisms and, using the maximum embolism area, determined the percentage of accumulated embolisms. We then created a table comparing the estimated water potential (described above) with the percentage of accumulated embolisms. Using this table, we identified the water potential values corresponding to 20% (*P*_20_), 50% (*P*_50_), and 80% (*P*_80_) embolism formation, defined as the water potential at which the respective percentage of embolisms was first observed. Finally, in Excel, we calculated the average *P*_20_, *P*_50_, and *P*_80_ values for each species and the standard error as the standard deviation divided by the root of the number of individuals.

To compare *P*_20_, *P*_50_, and *P*_80_ between species, we conducted an independent *t*‐test and analysis of covariance in JASP (https://jasp-stats.org/), using the values of each individual as replicates. Finally, we plotted the drought vulnerability curves using GraphPad Prism (version 10, GraphPad Software, San Diego, California, USA), applying a different color to each curve depending on the stem diameter.

### Results

**Figure 5:**
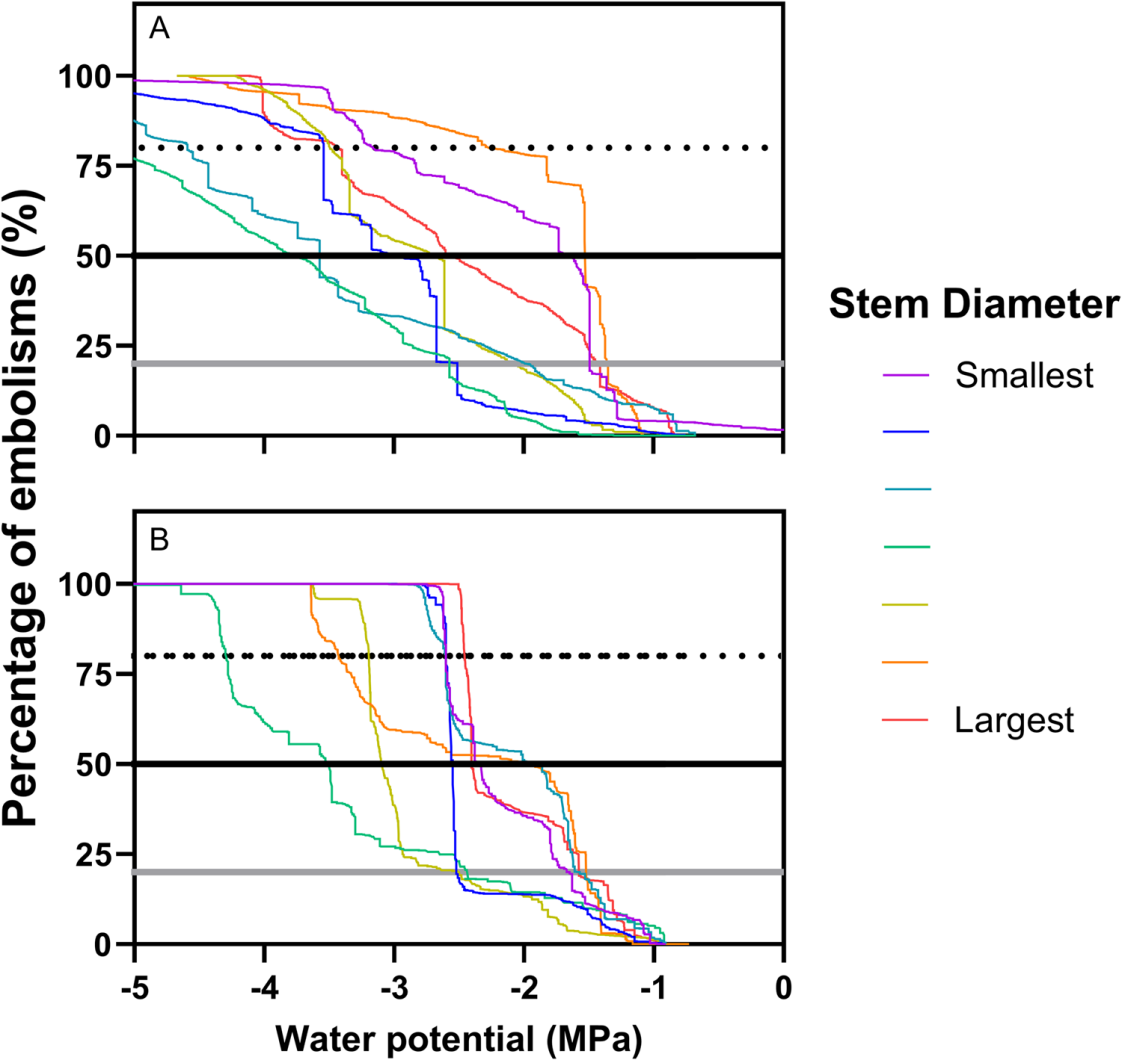
Vulnerability curves for (A) *Rhus integrifolia* and (B) *Nicotiana glauca*. The water potential at which each individual reaches 20% (*P*_20_), 50% (*P*_50_), and 80% (*P*_80_) of hydraulic conductivity loss in stems is indicated by a gray line, black line, and dotted black line, respectively. The different colors represent the varying stem diameters where the xylem was exposed, following a rainbow pattern from smallest to largest diameter (purple to red; see Appendix S5 for more details).

The native *R. integrifolia* had a *P*_50_ value of −2.67 ± 0.32 MPa (mean ± standard error; Figure 5A), while the exotic species *N. glauca* had a *P*_50_ of −2.52 ± 0.23 MPa (Figure 5B, Appendix S6). A *t*‐test showed no significant difference in *P*_50_ between the two species (*t* = −0.360, *df* = 12, *P* = 0.842). The shape of the curves suggests that *N. glauca* exhibits a faster embolism formation. In contrast, the curves for *R. integrifolia* showed a more gradual slope, indicating a progressive loss of conductivity without abrupt changes.

Consequently, the water potential at which the individuals reached 80% embolism (*P*_80_) was −3.67 ± 0.44 MPa for *R. integrifolia* and −3.03 ± 0.31 MPa for *N. glauca*. This suggests that when using *P*_80_ as an indicator of drought resistance, *R. integrifolia* is more resistant than *N. glauca*. In contrast, *P*_20_ values were slightly more positive in *R. integrifolia* (*P*_20_ = −1.91 ± 0.24 MPa) than in *N. glauca* (*P*_20_ = −1.96 ± 0.24 MPa). Despite these differences, the *t*‐test showed no significant difference in *P*_80_ (*t* = 1.467, *df* = 12, *P* = 0.168) or in *P*_20_ (*t* = −0.204, *df* = 12, *P* = 0.842) between the two species.

We also measured the stem diameter and length for each individual (Figure 5, Appendix S6). There was no significant difference in *P*_20_, *P*_50_, and *P*_80_ between species when stem diameter and stem length were included as covariates in an analysis of covariance.

## CONCLUSIONS

Modifications to the optical method of Brodribb et al. (2017) allowed us to obtain the plant water potential at which 50% of the xylem was embolized (*P*_50_) in *N. glauca* and *R. integrifolia* using accessible and economical materials. The program developed to photograph embolism formation in the xylem also enabled us to capture individual measurements from multiple specimens, highlighting the intraspecific variability in *P*_50_ values and the shape of the vulnerability curves—an aspect considered relevant in previous literature (Sperry and Saliendra, 1994; Sperry et al., 2012; Torres‐Ruiz et al., 2017). The program also enabled easy viewing and focusing of photos as needed. By following the steps outlined by Lucani and Brodribb (2023), we were able to quantify the embolisms and obtain the curves that allowed us to calculate *P*_50_.

This protocol is cost‐effective compared to other optical methods, as the total cost for the materials needed to simultaneously measure four stems is approximately $224.43 USD. This price falls between more expensive conventional scanners and a single smartphone (Table 1), with the added advantage of allowing simultaneous measurements of four individuals. The cost of a single stereoscopic microscope is nearly double the cost of all our combined materials (Table 1). Raspberry Pi cameras (https://www.raspberrypi.com/) can be equally expensive because their cost excludes the optical zoom elements, the Raspberry Pi, as well as the additional materials needed to secure the stems to the camera. Moreover, most of our materials are widely available worldwide through commercial platforms such as Amazon. The protocol requires only a darkened room and compact equipment that fits into a small box, ensuring portability. This accessibility, combined with the simplicity of application, makes the method highly field‐deployable and advantageous over other optical methods for the global scientific community. Our variation of the optical method is highly scalable, allowing for easy connection of a larger or smaller number of microscopes as needed, with only minimal software adjustments required. To further minimize the cost of our protocol, the next critical step is lowering the cost of the method to quantify the stem water potential, as the pressure chamber is very expensive in comparison with the rest of the components. Future designs may incorporate new ideas to surrogate the stem water potential by the pressure chamber, as the mechanical simplicity and resistance of the current pressure chambers are an excellent standard due to their durability and ease of operation, but with the trade‐off of a high economic cost.

A deeper analysis of the species studied revealed that *R. integrifolia* and *N. glauca* have average *P*_50_ values of −2.67 MPa and −2.52 MPa, respectively (Figure 5). Although the difference between the species is not statistically significant, these results suggest that *R. integrifolia* may be slightly more resistant to drought than *N. glauca*. The *P*_50_ values for both species fall within the range reported for other arid‐zone species, such as those from Peru and Tasmania (Blackman et al., 2012). However, these values differ from those reported by Jacobsen et al. (2007), Pivovaroff et al. (2014, 2016), and Ennajeh et al. (2022) (Table 2). Such discrepancies may be attributed to variations in dehydration methods and the indirect approach used to assess embolism formation (percentage loss of conductivity). For instance, when a centrifuge is used as the dehydration method, vessel artifacts can arise because the stem size must be reduced to fit into the centrifuge chamber (Torres‐Ruiz et al., 2017); in contrast, the dry‐bench method employed by Jacobsen et al. (2007) and in our study does not impose such size restrictions. Moreover, *R. integrifolia* exhibits a longer maximum vessel length compared to *N. glauca* and other native species of the Californian shrubland (Jacobsen et al., 2007) (Appendix S5). This characteristic makes it more susceptible to open‐vessel artifacts, which can affect *P*_50_ measurements. Conversely, the optical method has its own limitations, as it visually detects embolism formation in superficial vessels of a small area but does not directly measure functional loss of conductivity. This distinction may lead to discrepancies when comparing *P*_50_ values across different studies (Pratt et al., 2020; Cardoso et al., 2022); therefore, these methodological differences must be carefully considered when interpreting and comparing *P*_50_ values from various references.

**Table 2:** Comparison of the water potential that causes 50% of hydraulic conductivity loss in stems (*P*_50_) of our studied species (*Rhus integrifolia* and *Nicotiana glauca*) and related species within the same genus (*Rhus ovata* and *Nicotiana tobaccum*) as obtained by other authors.

Species	*P*_50_	Dehydration method	References^a^
*Rhus integrifolia*	−1.8	Bench‐dry	Jacobsen et al. (2007)
*Rhus ovata*	−1.4	Bench‐dry	Jacobsen et al. (2007)
	−1.9	Centrifugation	Pivovaroff et al. (2014)
	−0.3	Centrifugation	Pivovaroff et al. (2016)
*Nicotiana glauca*	−3.7	Centrifugation	Pivovaroff et al. (2014)
*Nicotiana tobaccum*	−1.18	Bench‐dry	Ennajeh et al. (2022)

^a^ In all of these references, the percent loss of conductivity (PLC) was calculated as an indirect method for estimating the formation of embolisms.

In addition, the differences in hydraulic strategies between the species may not be fully explained by *P*_50_ alone. The shape of the curves suggests that hydraulic failure in *N. glauca* occurs rapidly after crossing a threshold between −2 and −3 MPa, whereas in *R. integrifolia* hydraulic conductivity is gradually lost.

We anticipated differences in *P*_50_ between the species, as *N. glauca* is a fast‐growing exotic species, while *R. integrifolia* is a slow‐growing native species. Díaz de León Guerrero et al. (2020) reported that native species of Californian Mediterranean vegetation (including *R. integrifolia*) have hydraulic and morphological attributes more strongly related to resource conservation than exotic species (including *N. glauca*). As expected, the native species *R. integrifolia* had more negative *P*_50_ values, indicating greater drought resistance. The shape of the curve also supports this conclusion, leading us to advocate for including additional parameters such as curve slope, along with synthetic estimators like *P*_20_, *P*_50_, and *P*_80_, to better interpret hydraulic behavior across species. Therefore, our variation of the optical method allowed us to describe the intra‐ and inter‐individual patterns of variability in hydraulic conductivity during drought. However, we only analyzed two species and recommend expanding *P*_50_ measurements to a broader range of species, varieties, and growth forms to validate its utility.

As our alternative optical method for quantifying *P*_50_ and vulnerability curves is extremely cost‐effective and accessible to researchers worldwide, we believe that further promotion and refinement of this method will deepen our understanding of plant variability in response to drought. In a world where plant sciences can contribute to solving societal and global problems, this method could be a valuable tool for foresters, ecologists, and agronomists in assessing the importance of drought resistance in ecosystem and agricultural management.

## AUTHOR CONTRIBUTIONS

R.M.A. and G.G.R. conceived the idea. M.A.A.A. designed the program. M.A.A.A. and E.L. contributed to the electrical and physical engineering part of the protocol. R.M.A. and G.G.R. contributed to the biological part of the protocol. G.G.R. and R.M.A. collected and analyzed the data. R.M.A., G.G.R., and M.A.A.A. wrote the manuscript. All authors approved the final version of the manuscript.

## Supporting information


**Appendix S1.** Temperature of the LED lamp and temperature and light intensity of the part of the stem receiving direct light (“stem with light”) in four individuals of *Rhus integrifolia* in the experimental setup, with the average and standard error (SE) indicated.

**Appendix S2.** Python source code for adjusting the image focus and lighting.

**Appendix S3.** Python source code for image acquisition.

**Appendix S4.** Data used to plot the vulnerability curves for the seven individuals of *Rhus integrifolia* and seven individuals of *Nicotiana*. The calculation and estimation of water potential and percentage of accumulated embolisms before obtaining the vulnerability curves and the water potential in which the individuals of each species presented 20% (*P*_20_), 50% (*P*_50_), and 80% (*P*_80_) of embolisms are also provided.

**Appendix S5.** Values of maximum xylem vessel length (*L*_max_) of six individuals of *Nicotiana glauca* and six individuals of *Rhus integrifolia*, with the average and standard error (SE) indicated for each species.

**Appendix S6.** Species, individual number, diameter, length of cut stem, and water potential in which the individuals presented 20% (*P*_20_), 50% (*P*_50_), and 80% (*P*_80_) of embolisms.

## Figures and Tables

**Figure A1:**
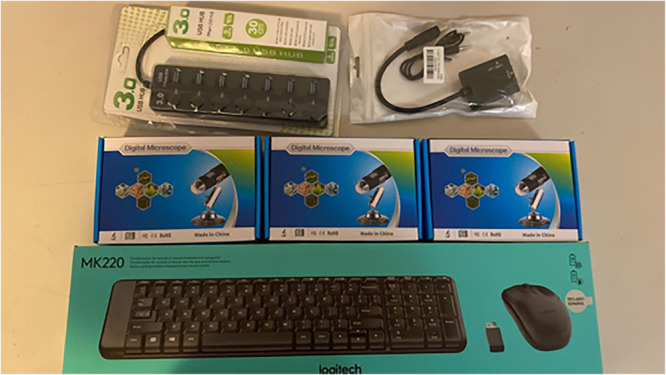
Materials used to measure percent of cavitation accumulated.

**Figure A2:**
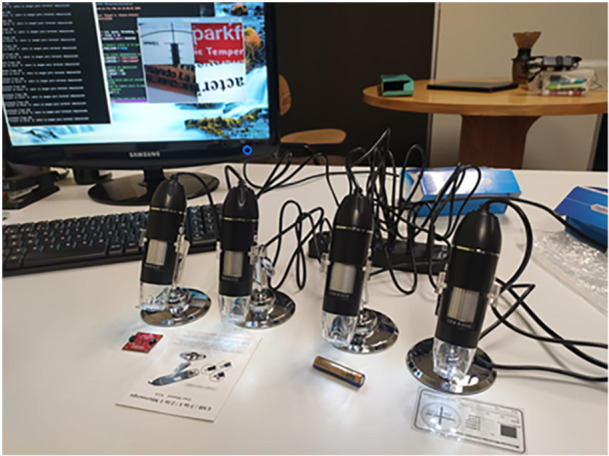
USB microscopes used.

## Data Availability

Data of all experiments are provided in the Supporting Information. The Python Program is available at: https://github.com/miguel-aalonso/lowcost_P50.

## Appendix 1 Detailed protocol for preparing plants and obtaining water potential and percent embolism accumulation with the simplified optical method.

The protocol, including links to the products, is also available at: https://github.com/miguel-aalonso/lowcost_P50

**A. Parts list**

- **Measurement of water potential**:
  ∘ Scholander‐type pressure chamber (PMS 1000, PMS Instrument Company, Corvallis, Oregon, USA)
  ∘ Magnifying glass or stereoscopic microscope
  ∘ Resealable zip‐top bags
  ∘ Absorbent paper towels
- Measurement of cavitation events (Figure A1):
  ∘ Four generic USB microscopes ($19.66 USD per microscope). These microscopes are low‐cost and can be easily acquired from commercial venues (e.g., Amazon or Steren Latin America) (Figure A2).
  ∘ Computer monitor
  ∘ Keyboard with mouse
  ∘ Desktop computer or Raspberry Pi (https://www.raspberrypi.com/)
  ∘ External lights (LED lamps)
  ∘ Silver tape (duct tape)
  ∘ Microscope supports
  ∘ Plywood
  ∘ Wire clips and screws
  ∘ Cosmetology nail file
  ∘ Tensive Conductive Adhesive Gel (Parker Laboratories, Fairfield, New Jersey, USA)
  ∘ Required software:
  − ImageJ (https://imagej.net/ij/download.html)
  − Python (https://www.python.org/downloads/)

**B. Preparation of equipment**

- **Microscope setup**
  ∘ Acquire four microscope stands and four USB microscopes.
- **Mounting the equipment**
  ∘ Cut a piece of birch plywood to the dimensions 160 cm × 50 cm × ½ inch.
  ∘ Perforate the microscope bases and secure into the plywood.
  ∘ Use screws and wire clips to secure branches and stems to the plywood and to the microscope stands.
- **Lighting adjustment**
  ∘ Disable the integrated LED lights in the microscopes.
  ∘ Set up external diffused lighting using LED lanterns to reduce reflections on moist plant tissues.
- **Image acquisition software**
  ∘ Use the Python programs (source code provided in Appendices S2 and S3) to capture photos from all four microscopes every four minutes.
  ∘ Ensure the programs are set for simultaneous image display and calibration, with time‐stamping to allow for later analysis.
- **Microscope arrangement in the darkroom:**
  ∘ Set up the microscopes in a darkroom to minimize external light interference.
  ∘ Arrange the water potential measurement station outside the darkroom.

**C. Procedure for measurement of** ***P***_**50**_

- **Collection of samples**
  ∘ Species: Select study species.
  ∘ Branch length: Approximately 50 cm for each sample.
  ∘ Rehydration: Rehydrate branches for 24 hours in buckets of water covered with black bags.
- **Xylem exposure**
  ∘ Use a cosmetology nail file to remove the bark and expose the xylem tissue. File away the hard outer bark and manually peel off the phloem using a small razor or fingernail until the white‐yellowish xylem is visible.
- **Preventing desiccation**
  ∘ Apply Tensive Conductive Adhesive Gel to the exposed xylem to prevent desiccation and enhance visibility under the microscope.
- **Imaging**
  ∘ Place the branches under the microscopes in the darkroom and begin image acquisition.
- **Water potential measurement**
  ∘ Bag the branches for 15 minutes after incision, with moist paper towels inside the bag.
  ∘ Use a pressure chamber for measuring water potential.
  ∘ Measure water potential every six hours for the first three days, then daily at 1200 hours.
- **Prepare vulnerability to cavitation curves**
  ∘ Follow the methodology outlined by Lucani and Brodribb (2023) using Fiji software (Schneider et al., 2012; https://imagej.net/software/fiji/) and Excel (Microsoft Corporation, Redmond, Washington, USA) to obtain the curves.
