# Supplementary material for: A low‐cost protocol for the optical method of vulnerability curves to calculate P 50
Source: Appl Plant Sci. 2025 Mar 31;13(2):e70004. doi: 10.1002/aps3.70004 (PMC12038744; doi:10.1002/aps3.70004)
Supplement: Supplementary file 1 — Appendix S1. Temperature of the LED lamp and temperature and light intensity of the part of the stem receiving direct light (“stem with light”) in four individuals of Rhus integrifolia in the experimental setup, with the average and standard error (SE) indicated. [file APS3-13-e70004-s004.docx]

**Appendix S1.** Temperature of the LED lamp and temperature and light intensity of the part of the stem receiving direct light (“stem with light”) in four individuals of *Rhus integrifolia* in the experimental setup, with the average and standard error (SE) indicated.^a^

|  | **LED lamp** | **Stem with light** | |
| --- | --- | --- | --- |
| **Individual** | **Temperature (°C)** | **Temperature** **(°C)^b^** | **Light intensity (mW)^b^** |
| 1 | 27.6 | 17.4 | 19.8 |
| 2 | 27.2 | 17.2 | 19.5 |
| 3 | 26.4 | 17.2 | 19.7 |
| 4 | 26.9 | 17.8 | 17.9 |
| Average | 27.02 | 17.4 | 19.2 |
| SE | 0.21 | 0.12 | 0.38 |

^a^The experiment was performed on 12 December 2024 at 10:33 a.m. local time (Ensenada, Baja California, Mexico).

**^b^**The room temperature and relative humidity were 17°C and 52%, respectively. The light intensity of the experimental setup far away from the LED lamp was 10.6 mW, and the light intensity in the area of the room far from the experiment setup was 7.7 mW. The room temperature and humidity were measured with a CO_2_ monitor (FD-CO2000-USB, Forensics Detectors, Rolling Hills Estates, California, USA). Surface temperatures were measured with an infrared electronic thermometer (DN-997, Blunt Bird, Dongguan, China). Light intensity was measured with an optical potential sensor (818 SL, Newport Corporation, Irvine, California, USA) and a digital multimeter (289, Fluke Corporation, Everett, Washington, USA).
