# Supplementary material for: A low‐cost protocol for the optical method of vulnerability curves to calculate P 50
Source: Appl Plant Sci. 2025 Mar 31;13(2):e70004. doi: 10.1002/aps3.70004 (PMC12038744; doi:10.1002/aps3.70004)
Supplement: Supplementary file 2 — Appendix S2. Python source code for adjusting the image focus and lighting. [file APS3-13-e70004-s006.docx]

**Appendix S2.** Python source code for adjusting the image focus and lighting.

Available at: <https://github.com/miguel-aalonso/lowcost_P50>

"""

DISCLAIMER:

This software is provided "as is", without any warranty of

any kind, express or implied, including but not limited to

the warranties of merchantability, fitness for a particular

purpose, and noninfringement. In no event shall the authors

or copyright holders be liable for any claim, damages, or

other liability, whether in an action of contract, tort, or

otherwise, arising from, out of, or in connection with the

software or the use or other dealings in the software.

This program was developed and tested using Python 3.10.14

on Raspberry Pi OS with Desktop, running on a Raspberry Pi

4B.

Due to the generic nature of low-cost USB microscopes

(cameras), they lack serial numbers or unique identifiers

for differentiation. To identify and enumerate them for

image acquisition, it is important to connect one device at

a time. Under Linux, devices will be numbered based on the

order in which they are connected. The connection of the

devices must be conducted before running this program.

This program should be run first. It does not capture images

but is designed to assist the user in positioning the

microscopes over the plant, as well as adjusting focus and

lighting before image acquisition.

CICESE, Ensenada, B.C.

Tue Sep 24 01:49:34 PM PDT 2024

"""

import numpy as np

import threading

import cv2

import os

import time

os.environ['DISPLAY'] = ':0'

# Each microscope is seen as two devices in /dev/video, one of them is real, the other is only camera metadata

cam_ids = [0,2,4,6]

SAVE_TIME = 5

# Image stack parameters

nm = len(cam_ids)

h, w = 240,320

c = 3

# Visualization parameters

cols = 2

rows = np.ceil(nm/cols).astype('uint8')

vis = np.zeros((rows*h,cols*w,c), dtype='uint8')

frame_num = 0

# Creates data directories

print('Building directory tree...')

os.makedirs('data', exist_ok=True)

for i in range(nm):

os.makedirs(f'data/microscope{i}', exist_ok=True)

class camThread(threading.Thread):

def __init__(self, camID):

threading.Thread.__init__(self, daemon = True)

self.camID = camID

self.cap_device = None

self.cap_state = False

def run(self):

print(f'Starting camera {self.camID}')

self.cam_init()

self.cam_preview()

def cam_init(self):

self.cap_device = cv2.VideoCapture(self.camID)

self.cap_device.set(3,h)

self.cap_device.set(4,w)

def cam_preview(self):

while True:

if self.cap_state == False:

try:

ret, frame = self.cap_device.read()

except:

print(f'intento fallido en {self.camID} retrying...')

if ret:

pos = self.camID//2

i = pos%cols

j = pos//cols

vis[j*h:(j+1)*h,

i*w:(i+1)*w,

:] = frame

else:

time.sleep(1)

def snapshot_thread():

frame_num = 0

print('Initializing capturing session...')

time.sleep(1)

while True:

start = time.time()

for thread in threads:

thread.cap_device.release()

thread.cap_state = True

print(f'Saving stack with frame number: {frame_num:05d}.')

for i, cam_id in enumerate(cam_ids):

cam = cv2.VideoCapture(cam_id)

if cam is None or not cam.isOpened():

print(f'Frame error in cam_id {cam_id}')

else:

for rep in range(5):

ret, frame = cam.read()

cv2.imwrite(f'data/microscope{i}/M{i}_{frame_num:05d}.png',frame)

cam.release()

for thread in threads:

thread.cam_init()

thread.cap_state = False

frame_num += 1

t_processing = time.time() - start

t_save = SAVE_TIME - t_processing

if t_processing > SAVE_TIME:

t_save = SAVE_TIME

time.sleep(t_save)

snapshot = threading.Thread(target=snapshot_thread, daemon=True)

threads = [camThread(cam_id) for cam_id in cam_ids]

for thread in threads:

thread.start()

fxy = 1

snapshot_running = False

cv2.namedWindow('Visualization', 16)

cv2.resizeWindow('Visualization',fxy*vis.shape[1],fxy*vis.shape[0])

while True:

overlay = vis.copy()

cv2.putText(img=overlay,

text=time.strftime("%d-%m-%Y %H:%M:%S", time.localtime()),

org=(5, 30),

fontFace=cv2.FONT_HERSHEY_TRIPLEX,

fontScale=1,

color=(0, 255, 0),

thickness=1)

cv2.imshow('Visualization', overlay)

key = cv2.waitKey(20)

if key == 27: # exit on ESC

cv2.destroyAllWindows()

exit()
