# Supplementary material for: A low‐cost protocol for the optical method of vulnerability curves to calculate P 50
Source: Appl Plant Sci. 2025 Mar 31;13(2):e70004. doi: 10.1002/aps3.70004 (PMC12038744; doi:10.1002/aps3.70004)
Supplement: Supplementary file 3 — Appendix S3. Python source code for image acquisition. [file APS3-13-e70004-s003.docx]

**Appendix S3.** Python source code for image acquisition.

Available at: <https://github.com/miguel-aalonso/lowcost_P50>

"""

DISCLAIMER:

This software is provided "as is", without any warranty of

any kind, express or implied, including but not limited to

the warranties of merchantability, fitness for a particular

purpose, and noninfringement. In no event shall the authors

or copyright holders be liable for any claim, damages, or

other liability, whether in an action of contract, tort, or

otherwise, arising from, out of, or in connection with the

software or the use or other dealings in the software.

This program was developed and tested using Python 3.10.14

on Raspberry Pi OS with Desktop, running on a Raspberry Pi

4B.

Due to the generic nature of low-cost USB microscopes

(cameras), they lack serial numbers or unique identifiers

for differentiation. To identify and enumerate them for

image acquisition, it is important to connect one device at

a time. Under Linux, devices will be numbered based on the

order in which they are connected. The connection of the

devices must be conducted before running this program.

This code handles image acquisition and should be run after

the microscopes' lighting and focus have been properly

adjusted.

CICESE, Ensenada, B.C.

Tue Sep 24 03:15:36 PM PDT 2024

"""

from threading import Thread

import numpy as np

import time

import cv2

import os

SAVE_TIME = 180 #Save image frame every N seconds

DEBUG = False

os.environ['DISPLAY'] = ':0'

# Each microscope is seen as two devices in /dev/video, one of them is real, the other is only camera metadata

cam_ids = [0,2,4,6]

# Image stack parameters

nm = len(cam_ids)

h, w = 480,640

c = 3

# Visualization parameters

cols = 2

rows = np.ceil(nm/cols).astype('uint8')

stack = np.zeros((nm,h,w,c), dtype='uint8')

vis = np.zeros((rows*h,cols*w,c), dtype='uint8')

# Creates data directories

os.makedirs('data', exist_ok=True)

for i in range(nm):

os.makedirs(f'data/microscope{i+1}', exist_ok=True)

def update_vis():

while True:

start = time.time()

for i,cam_id in enumerate(cam_ids):

cam = cv2.VideoCapture(cam_id)

aug = np.zeros((h,w,c))

if cam is None or not cam.isOpened():

print(f'Frame error in cam_id {cam_id}')

frame = np.zeros((h,w,c))

else:

for rep in range(10):

ret, frame = cam.read()

aug += frame

if rep == 9:

frame = aug/10

#print('Average Frame')

if ret:

cam.release()

frame = frame

stack[i] = frame

#Updates visualization grid

y = i//cols

x = i%cols

vis[y*h:(y+1)*h,

x*w:(x+1)*w,

:] = frame

if DEBUG:

print(f'Frame time: {time.time()-start:.2f} s')

def save_stack():

print('Initializing capturing session...')

time.sleep(1)

frame_num = 0

while True:

start = time.time()

for i,img in enumerate(stack):

cv2.imwrite(f'data/microscope{i+1}/M{i+1}_{frame_num:05d}.png',img)

print(f'Stack saved with frame number: {frame_num:05d}, Timestamp: {time.strftime("%d-%m-%Y %H:%M:%S", time.localtime())}.')

frame_num += 1

t_processing = time.time() - start

t_save = SAVE_TIME - t_processing

if t_processing > SAVE_TIME:

t_save = SAVE_TIME

time.sleep(t_save)

if DEBUG:

print(f'Elapsed time: {time.time() - start:.2f} s, Processing_time: {t_processing:.2f} s')

cam_thread = Thread(target=update_vis, daemon=True)

snapshot_thread = Thread(target=save_stack, daemon=True)

cam_thread.start()

fxy = 0.5

x,y = int(fxy*vis.shape[1]), int(fxy*vis.shape[0])

snapshot_running = False

cv2.namedWindow('Visualization', 16)

cv2.resizeWindow('Visualization',x,y) #Window Size

while True:

overlay = vis.copy()

cv2.putText(img=overlay,

text=time.strftime("%d-%m-%Y %H:%M:%S", time.localtime()),

org=(5, 30),

fontFace=cv2.FONT_HERSHEY_TRIPLEX,

fontScale=1,

color=(0, 255, 0),

thickness=1)

cv2.imshow('Visualization', overlay)

key = cv2.waitKey(20)

if key == 27: # exit on ESCa

break

elif key == ord('a'): # Start capturing

if snapshot_running == False:

snapshot_thread.start()

snapshot_running = True

else:

print('Capturing session is already on.')

cv2.destroyAllWindows()

exit()
