# Supplementary material for: A low‐cost protocol for the optical method of vulnerability curves to calculate P 50
Source: Appl Plant Sci. 2025 Mar 31;13(2):e70004. doi: 10.1002/aps3.70004 (PMC12038744; doi:10.1002/aps3.70004)
Supplement: Supplementary file 5 — Appendix S5. Values of maximum xylem vessel length (L max) of six individuals of Nicotiana glauca and six individuals of Rhus integrifolia, with the average and standard error (SE) indicated for each species. [file APS3-13-e70004-s002.docx]

**Appendix S5.** Values of maximum xylem vessel length (*L*_max_) of six individuals of *Nicotiana glauca* and six individuals of *Rhus integrifolia*, with the average and standard error (SE) indicated for each species.

| **Species** | **Individual** | ***L*_max_ (m)** |
| --- | --- | --- |
| *Nicotiana glauca* | 1 | 0.54 |
|  | 2 | 0.61 |
|  | 3 | 0.51 |
|  | 4 | 0.46 |
|  | 5 | 0.69 |
|  | 6 | 0.73 |
|  | **Average** | **0.59** |
|  | **SE** | **0.043** |
| *Rhus integrifolia* | 1 | 0.81 |
|  | 2 | 0.96 |
|  | 3 | 0.96 |
|  | 4 | 0.98 |
|  | 5 | 0.77 |
|  | 6 | 0.72 |
|  | **Average** | **0.86** |
|  | **SE** | **0.046** |
