# Supplementary material for: A low‐cost protocol for the optical method of vulnerability curves to calculate P 50
Source: Appl Plant Sci. 2025 Mar 31;13(2):e70004. doi: 10.1002/aps3.70004 (PMC12038744; doi:10.1002/aps3.70004)
Supplement: Supplementary file 6 — Appendix S6. Species, individual number, diameter, length of cut stem, and water potential in which the individuals presented 20% (P 20), 50% (P 50), and 80% (P 80) of embolisms. [file APS3-13-e70004-s005.docx]

**Appendix S6.** Species, individual number, diameter, length of cut stem, and water potential in which the individuals presented 20% (*P*_20_), 50% (*P*_50_), and 80% (*P*_80_) of embolisms.

| **Species** | **Individual** | **Diameter (mm)** | **Length (m)** | ***P*_20_** | ***P*_50_** | ***P*_80_** |
| --- | --- | --- | --- | --- | --- | --- |
| *Nicotiana glauca* | 1 | 12.71 | 1.17 | −1.65 | −2.34 | −2.60 |
|  | 2 | 15.94 | 1.60 | −1.57 | −1.86 | −2.60 |
|  | 3 | 13.98 | 1.30 | −2.52 | −2.55 | −2.60 |
|  | 4 | 16.72 | 1.39 | −2.44 | −3.52 | −4.30 |
|  | 5 | 16.37 | 1.61 | −1.52 | −1.91 | −3.43 |
|  | 6 | 16.17 | 1.66 | −2.50 | −3.09 | −3.20 |
|  | 7 | 17.45 | 2.05 | −1.58 | −2.4 | −2.46 |
| *Rhus integrifolia* | 1 | 7.47 | 0.93 | −1.49 | −1.64 | −3.20 |
|  | 2 | 13.14 | 1.11 | −2.57 | −3.76 | −5.21 |
|  | 3 | 16.87 | 1.02 | −2.51 | −2.93 | −3.54 |
|  | 4 | 15.22 | 1.03 | −1.97 | −3.57 | −4.59 |
|  | 5 | 13.82 | 1.26 | −2.09 | −2.68 | −3.49 |
|  | 6 | 17.36 | 1.34 | −1.35 | −1.53 | −2.26 |
|  | 7 | 13.73 | 1.68 | −1.42 | −2.56 | −3.44 |
